# Ten-Year Outcomes of Cervical Artery Dissection: A Retrospective Study in a Real-World Cohort

**DOI:** 10.3390/jcm14196836

**Published:** 2025-09-26

**Authors:** Marcello Lodato, Rodolfo Pini, Alessandra Porcelli, Enrico Gallitto, Andrea Vacirca, Mauro Gargiulo, Gianluca Faggioli

**Affiliations:** 1 Vascular Surgery Unit, IRCCS University Hospital Policlinico S. Orsola, 40138 Bologna, Italy; 2 Vascular Surgery, University of Bologna, DIMEC, 40138 Bologna, Italy

**Keywords:** cervical artery dissection, follow up, common carotid artery

## Abstract

**Introduction.** Cervical artery dissection (CAD) is a rare condition, being one of the leading causes of stroke in patients under the age of 45, with a reported prevalence of up to 20%. The management of CAD remains controversial due to its rarity and the lack of large-scale randomized controlled trials. The aim of this study was to report the long-term outcomes of CAD in a real-world setting. **Methods.** This retrospective, observational, single-center study included patients diagnosed with CAD between 2010 and 2019 (approval number: 153/2015/U/Oss/AOUBo). Clinical presentation, risk factors, and medical therapies were prospectively analyzed. Management strategies included both medical and interventional approaches. Follow-up consisted of annual clinical visits and carotid duplex ultrasound (DUS), with telephone interviews every six months. The primary endpoint was defined by the overall long-term stroke/death rate and in relation to the type of medical treatment, localization of the dissection and clinical manifestations. **Results.** A total of 62 patients were included, predominantly male (65%) with a mean age of 58 (±2) years. Thirteen dissections (21%) were trauma-related. CAD locations included the common carotid artery in 6 cases (10%), extracranial internal carotid artery in 29 (46%), intracranial internal carotid artery in 9 (14%), and vertebral artery in 16 (25%). One patient (2%) had dissections in both the extracranial internal carotid and vertebral arteries, and another (2%) in both the vertebral and basilar arteries. Bilateral dissections were observed in 5 patients (8%). Ischemic manifestations occurred in 43 patients (68%): 10 transient ischemic attacks (16%), 17 minor strokes (27%), and 16 major strokes (25%), with ischemic lesions on cerebral CT in 31 cases (72%). Fifty-eight (93%) patients were treated medically (anticoagulants and/or antiplatelets), while 4 patients (7%) underwent surgical or endovascular intervention. The mean follow-up was 81 ± 35 months. During this period, 2 patients (4%) experienced stroke and 15 (24%) died. The estimated 10-year survival rate was 71%, and the 10-year stroke/death-free survival rate was 70%. Among medically treated patients, the 10-year stroke/death-free survival was 86% for those on anticoagulation and 67% for those on antiplatelet therapy (*p* = 0.1). Patients presenting with ischemic symptoms had a lower estimated 10-year stroke/death-free survival rate compared to those with non-ischemic presentations (61% vs. 69%, *p* = 0.7). Patients with dissection of the common carotid artery had a significantly lower estimated 10-year stroke/death-free survival rate (25%), compared to dissections in other cervical arteries (*p* = 0.001). **Conclusions.** In this real-world, single-center experience, cervical artery dissection was associated with a favorable long-term prognosis in most cases, especially among patients managed conservatively with medical therapy. Stroke and mortality rates were relatively low during extended follow-up. Although no statistically significant difference was observed between anticoagulation and antiplatelet therapy, the trend favored anticoagulation for stroke/death-free survival. Patients with CCA dissections had significantly worse 10-year stroke/death-free survival compared to those with dissections in other cervical arteries.

## 1. Introduction

Cervical artery dissection (CAD) is a relatively rare but important cause of stroke, particularly in young and middle-aged adults [1]. CAD accounts for approximately 2% of all ischemic strokes, but up to 20% in young and middle-aged adults (<45 years), with an estimated annual incidence of 2.6–3.0 per 100,000 for carotid dissections and 1.0–1.5 per 100,000 for vertebral dissections [2]. Cervical artery dissection can arise spontaneously or following trauma, with important differences in clinical profile and prognosis. Spontaneous dissections are often associated with predisposing conditions such as fibromuscular dysplasia, connective tissue abnormalities, or systemic arteriopathies, whereas trauma-related dissections may result from even minor mechanical injury including sports activities, chiropractic manipulation, or whiplash. Less frequently, inflammatory or infectious mechanisms have been implicated. This heterogeneity in etiology underscores the multifactorial nature of CAD and may contribute to variability in clinical presentation and outcomes [2]. Although often self-limiting, CAD can lead to serious neurological events and remains one of the leading causes of ischemic stroke in patients under 45 years of age [3,4]. Prompt diagnosis and effective management are therefore essential to prevent stroke recurrence and long-term disability.

CAD typically involves a tear in the intimal layer of a cervical artery—most commonly the internal carotid or vertebral artery—leading to the formation of an intramural hematoma or pseudoaneurysm. This can result in arterial stenosis, occlusion, or embolization, causing cerebral ischemia. Carotid duplex ultrasound can be used as the first-level approach to investigate extracranial carotid dissection with a high sensitivity. Either computed tomography angiography (CTA), magnetic resonance imaging (MRI), or magnetic resonance angiography (MRA) are recommended as diagnostic test. MRA, along with a T1 axial cervical MRI with fat saturation technique, has high sensitivity and specificity in detecting intramural hematoma. CTA can show both the intramural hematoma and the double lumen sign (true and false lumen) with high sensitivity and specificity. The clinical presentation is variable and may include local symptoms such as neck pain, headache, Horner’s syndrome, or neurological deficits due to cerebral ischemia. The heterogeneity of symptoms, often nonspecific, can delay diagnosis, which underscores the importance of high clinical suspicion in young stroke patients.

Despite growing clinical recognition and improved imaging techniques, the optimal treatment strategy for CAD remains a matter of debate. Medical therapy—either with antiplatelet agents or anticoagulants—is the mainstay of treatment, aimed at preventing thromboembolic complications. However, the choice between these two approaches is not standardized, as direct comparative data are limited and randomized controlled trials have yielded inconclusive results. Previous studies in literature found no significant difference between antiplatelet and anticoagulant therapy in preventing recurrent stroke, leading to considerable variation in practice across centers [5,6].

The CADISS trial, one of the few prospective randomized studies comparing antiplatelet and anticoagulant therapies in patients with extracranial carotid or vertebral dissections, found no statistically significant difference in stroke recurrence between the two groups [5]. Nonetheless, the trial was underpowered to detect small but clinically relevant differences and did not address intracranial dissections or long-term outcomes. As such, the decision regarding antithrombotic strategy is often left to clinician discretion, influenced by the patient’s clinical presentation, risk factors, and imaging findings. Moreover, the safety profiles of these therapies differ—anticoagulation may carry a higher risk of hemorrhagic transformation, while antiplatelet therapy may be less effective in cases with extensive intraluminal thrombus—further complicating management decisions.

Endovascular and surgical interventions are generally reserved for select cases, such as those with persistent symptoms despite optimal medical therapy, hemodynamic compromise, or rapidly worsening neurological deficits [7,8]. Interventional approaches may involve stenting of the dissected segment or surgical bypass in rare, complex cases. These strategies are typically reserved for high-risk patients or those with contraindications to medical therapy. However, their use is largely based on case reports and small series, without clear consensus or guideline recommendations, and long-term data on procedural durability and complication rates remain sparse.

Given the low incidence of CAD, most published data come from small cohorts or registries, often with heterogeneous populations and follow-up protocols. As a result, long-term outcomes of patients with CAD—particularly in relation to clinical presentation, dissection location, and treatment strategy—remain underreported and poorly understood. This lack of robust, long-term evidence hampers the ability to develop standardized management algorithms and identify prognostic subgroups who may benefit from tailored approaches.

In this context, this retrospective, single-center study aims to report the ten-year outcomes of this rare condition. We provide a decade of follow-up in a real-world cohort of patients diagnosed with CAD, analyzing stroke and mortality outcomes in relation to clinical presentation, type of medical treatment, and anatomical location of the dissection. Our findings aim to offer additional insights into the natural history of CAD and to inform treatment strategies in clinical practice.

## 2. Materials and Methods

### 2.1. Study Design and Population

This was a retrospective, observational, single-center study that included consecutive patients diagnosed with cervical artery dissection between January 2010 and December 2019. This 10-year time frame was selected to ensure both an adequate sample size and sufficient duration of follow-up for long-term outcome analysis. The study was conducted at a tertiary referral center with expertise in vascular and neurovascular pathology. It was approved by the local ethics committee (approval number: 153/2015/U/Oss/AOUBo), and all patients provided written informed consent for inclusion in the registry and subsequent data analysis. The study was performed in accordance with the Declaration of Helsinki and current ethical regulations governing observational research in clinical practice.

### 2.2. Diagnostic Criteria

The diagnosis of CAD was established based on typical imaging findings, in the context of an appropriate clinical presentation. Accepted radiological criteria included the presence of an intimal flap, double lumen, mural hematoma, pseudoaneurysm formation, or long tapered luminal stenosis or occlusion. These features were evaluated by neuroradiologists and vascular surgeons with experience in cerebrovascular disease. Imaging modalities employed included computed tomography angiography (CTA), magnetic resonance angiography (MRA), and duplex ultrasonography (DUS). In select cases where diagnostic uncertainty remained or intervention was being considered, digital subtraction angiography (DSA) was performed for confirmation. The diagnosis was established at the time of first clinical presentation and reviewed retrospectively for consistency by a multidisciplinary team.

### 2.3. Data Collection

Patient data were obtained from the institutional electronic medical records and vascular database. Baseline data included age, sex, cardiovascular risk factors (such as hypertension, smoking, hyperlipidemia, diabetes mellitus, and previous cardiovascular events), and potential predisposing conditions (e.g., trauma within 2 weeks, connective tissue disorders). Clinical presentation was classified into ischemic (stroke or transient ischemic attack) and non-ischemic symptoms (such as headache, neck pain, or Horner’s syndrome). Minor and major strokes were defined according to the NIHSS classification [9]. Neurological symptoms were confirmed by specialist evaluation, and ischemic events were documented with cerebral imaging (CT or MRI).

Anatomical localization of dissections was categorized into: common carotid artery, extracranial internal carotid artery, intracranial internal carotid artery, vertebral artery, and basilar artery. Multivessel and bilateral involvement were recorded. Imaging reports were cross-referenced to ensure accuracy of classification.

Therapeutic strategies, both medical and interventional, were documented in detail. Medical therapies included type and duration of antithrombotic treatment. In patients who underwent endovascular or surgical procedures, data on the type of procedure, indication, and complications were collected.

### 2.4. Treatment Strategies

The choice of treatment modality was individualized and made at the discretion of the treating neurologist in consultation with vascular surgeons and neuroradiologists. Initial decisions were influenced by the patient’s clinical status, imaging findings, anatomical location of dissection, and perceived risk of embolic events. Medical therapy consisted of antiplatelet agents (e.g., aspirin 100 mg daily, clopidogrel 75 mg daily or dual antiplatelet therapy), anticoagulants (low-molecular-weight heparin or oral vitamin K antagonists with target INR 2–3), or a sequential combination. Some patients initially received anticoagulation followed by a switch to antiplatelet therapy during follow-up.

The decision between anticoagulation and antiplatelet therapy was primarily individualized according to clinical and imaging features. Anticoagulation was generally favored in patients with imaging findings suggestive of higher embolic potential, such as the presence of a larger intraluminal thrombus, severe luminal stenosis, or pseudoaneurysm formation. Conversely, antiplatelet therapy was more frequently prescribed in patients with milder dissections, lower estimated risk of embolization, or when anticoagulation was contraindicated due to increased bleeding susceptibility.

Patients presenting with high-grade stenosis or occlusion, recurrent events despite therapy, or contraindications to anticoagulation were considered for invasive management. Interventions included carotid artery stenting or, in selected cases, carotid endarterectomy. All procedures were performed by experienced interventional radiologists or vascular surgeons, and procedural details and periprocedural outcomes were recorded.

### 2.5. Follow-Up

All patients were enrolled in a structured follow-up program. Clinical assessments were performed in dedicated outpatient clinics at 3 months, 6 months, 12 months, and annually thereafter. Carotid duplex ultrasonography (DUS) was performed at each visit to assess vascular patency, residual stenosis, and anatomical evolution. Additional imaging (CTA or MRA) was performed in patients with new neurological symptoms, suspected restenosis, or treatment failure.

To ensure comprehensive follow-up, patients were contacted via telephone every 6 months to assess treatment adherence, changes in clinical status, and occurrence of adverse events. If telephone contact failed or the patient was lost to follow-up, vital status was verified through national health records where available.

### 2.6. Study Endpoints

The primary endpoint of the study was the composite of any stroke (ischemic or hemorrhagic) and all-cause mortality during the follow-up period. Stroke events were confirmed by neurologic examination and imaging. Secondary endpoints included stroke/death-free survival stratified by clinical presentation (ischemic vs. non-ischemic), treatment modality (antiplatelet vs. anticoagulation), and anatomical location of the dissection (e.g., common carotid artery vs. other sites).

### 2.7. Statistical Analysis

All statistical analyses were conducted using SPSS^®^ software, version 21.0 (IBM Corp., Armonk, NY, USA). Continuous variables were tested for normality using the Shapiro–Wilk test and are presented as mean ± standard deviation (SD) or median with interquartile range (IQR), as appropriate. Between-group comparisons were performed using the Student’s *t*-test or the Mann–Whitney U-test. Categorical variables were reported as frequencies and percentages and compared using the Chi-square test or Fisher’s exact test.

Time-to-event outcomes (stroke/death-free survival and overall survival) were assessed using Kaplan–Meier curves, and comparisons across groups were made with the log-rank test. Patients who were lost to follow-up were censored at the time of their last contact. A two-sided *p*-value of ≤0.05 was considered statistically significant for all analyses.

Given the retrospective design and limited sample size, no multivariable regression modeling or propensity score adjustment was performed.

## 3. Results

A total of 62 patients diagnosed with CAD between January 2010 and December 2019 were included in the analysis. The cohort was predominantly male (n = 40, 65%) with a mean age of 58 (±2) years. Thirteen patients (21%) had trauma-related dissections, whereas the remaining 49 (79%) patients had spontaneous dissections. Demographics, cardiovascular risk factors and preoperative comorbidities are reported in Table 1.

### 3.1. Anatomic Distribution of Dissections

Dissections predominantly involved the extracranial internal carotid artery (EICA), which accounted for 30 cases (48%). Other sites of dissection included the vertebral artery (VA) in 16 patients (26%), the intracranial internal carotid artery (IICA) in 9 patients (14%), and the common carotid artery (CCA) in 6 patients (10%). A single dissection of the basilar artery was recorded (2%). Additionally, five patients (8%) presented with bilateral dissections. Two patients (4%) had dissections involving multiple arteries: one involving both the EICA and VA, and another involving both the VA and basilar artery. The full distribution is detailed in Table 2.

### 3.2. Clinical Presentation

Forty-three patients (69%) presented ischemic symptoms: transient ischemic attacks (TIAs) in 10 (16%) patients, minor strokes in 17 (27%) patients, major strokes in 16 (25%) patients.

Neuroimaging (primarily CT scans) confirmed the presence of ischemic lesions in 31 of these 43 patients (72%). The remaining 19 patients (31%) presented with non-ischemic symptoms such as headache, neck pain, or Horner’s syndrome. Table 3 reports the clinical manifestations of all the patients.

**Figure 1:**
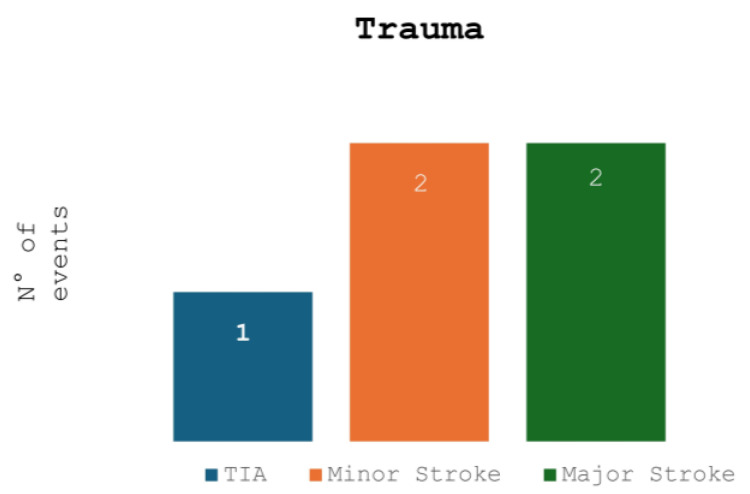
Trauma-related dissections and ischemic symptoms.

**Figure 2:**
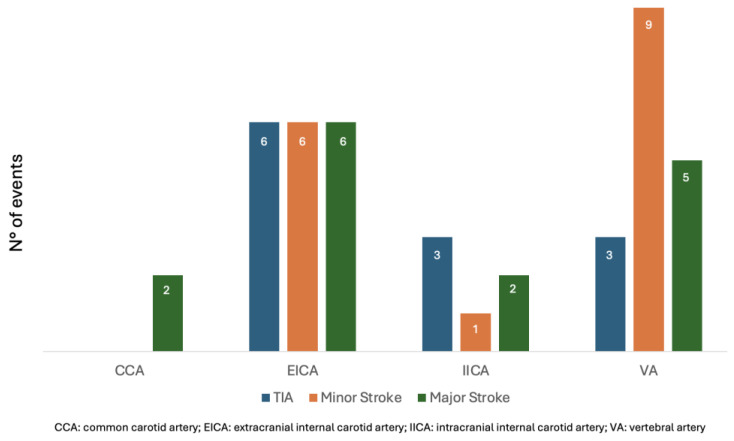
Ischemic symptoms compared to the location of dissections.

Figure 1 and Figure 2 compare the ischemic symptoms with dissections trauma-related and their localization.

### 3.3. Management Strategies

Fifty-eight (92%) patients were managed conservatively with medical therapy: 25 (43%) patients received anticoagulation (primarily vitamin K antagonists or low-molecular-weight heparin), 28 (48%) patients received antiplatelet agents (most commonly aspirin or clopidogrel), 5 (9%) patients received a dual antiplatelet therapy.

Four patients (7%) underwent interventional procedures: one carotid endarterectomy (2%) and 3 stent placement (5%), due to contraindications to medical therapy, progressive symptoms, or hemodynamic instability.

No cross-over between treatment strategies was observed during follow-up. Patients treated with stents received dual antiplatelet therapy for 6 months, while the duration of therapy for those treated medically was standardized at 3 months. Treatment was well tolerated in all cases.

### 3.4. Follow-Up Outcomes

The median duration of follow-up was 78 (IQR 54–106) months. During this period, 2 patients (4%) experienced recurrent ischemic stroke, both occurring within the first 12 months following the index event. One of these patients had a dissection involving the vertebral artery and was initially treated with antiplatelet therapy. The other had a common carotid artery dissection and was managed with anticoagulation. Both patients died from these recurrent ischemic strokes.

Fifteen patients (24%) died during follow-up. Causes of death included cardiac complications (n = 5), cancer (n = 4), stroke (n = 2), and other non-neurological causes (n = 4). No deaths were directly attributable to complications of dissection beyond the first year post-diagnosis.

**Figure 3:**
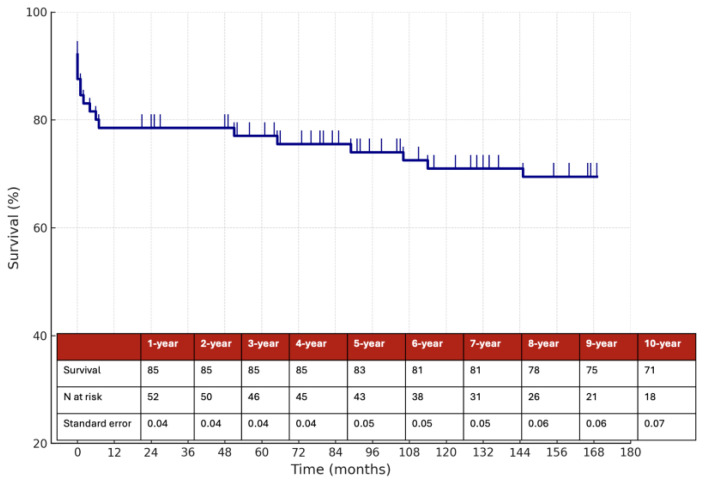
Estimated 10-year survival rate.

**Figure 4:**
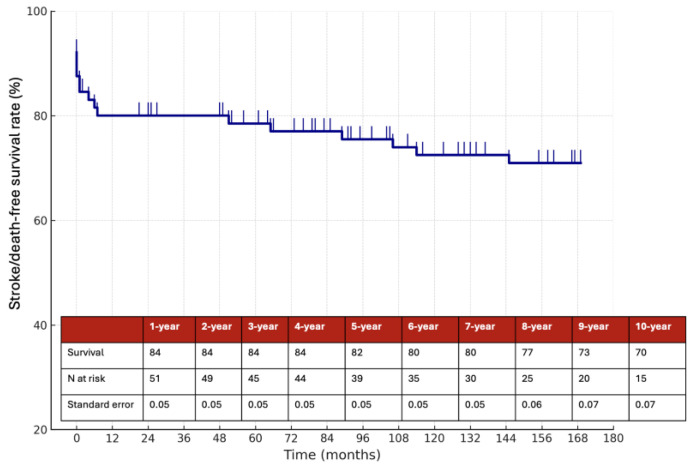
Estimated 10-year stroke/death-free survival rate.

**Figure 5:**
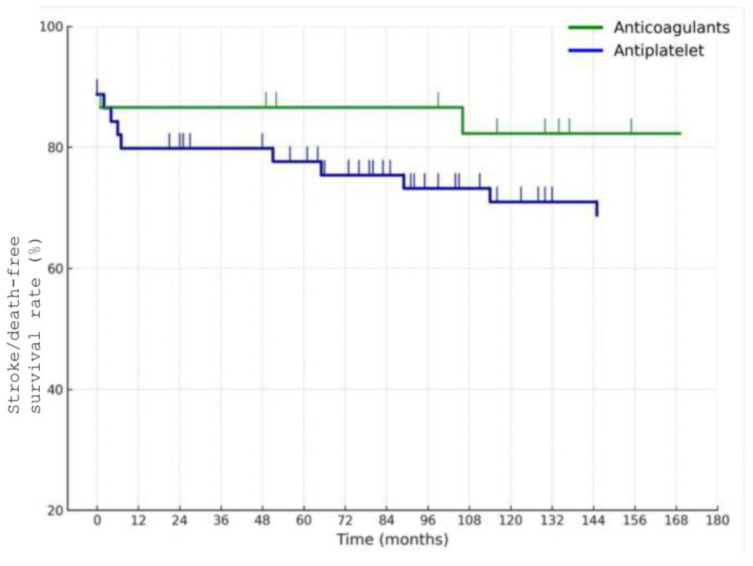
Estimated 10-year stroke/death-free survival rate in patients treated medically.

The estimated 10-year overall survival rate was 71%, as illustrated in Figure 3, while the 10-year stroke/death-free survival rate was 70% (Figure 4). Among the 58 patients managed medically, the 10-year stroke/death-free survival was 86% in the anticoagulation group and 67% in the antiplatelet group (Figure 5), although this difference did not reach statistical significance (*p* = 0.1).

**Figure 6:**
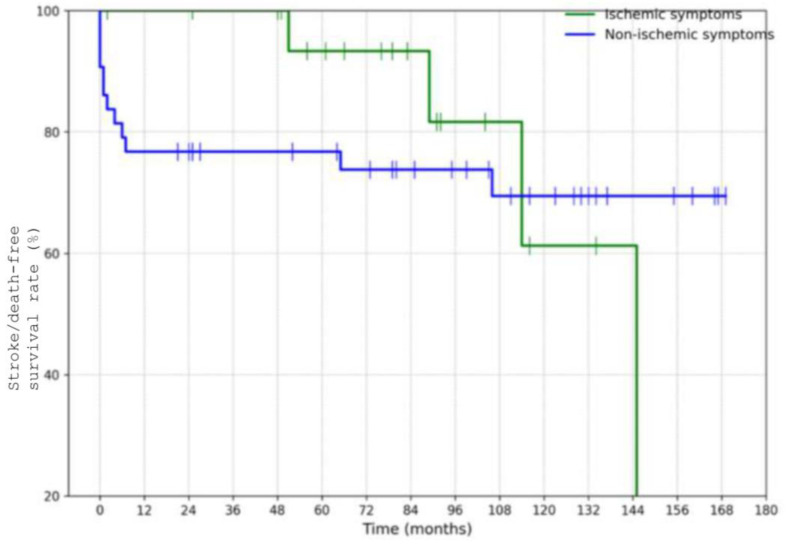
Estimated 10-year stroke/death-free survival rate in patients with ischemic manifestations.

Patients with ischemic presentation at baseline had lower 10-year stroke/death-free survival (61%) than those with non-ischemic symptoms (69%), although this was not statistically significant (*p* = 0.7) (Figure 6). This trend suggests that initial ischemic injury may be a marker of more aggressive disease course or embolic potential.

**Figure 7:**
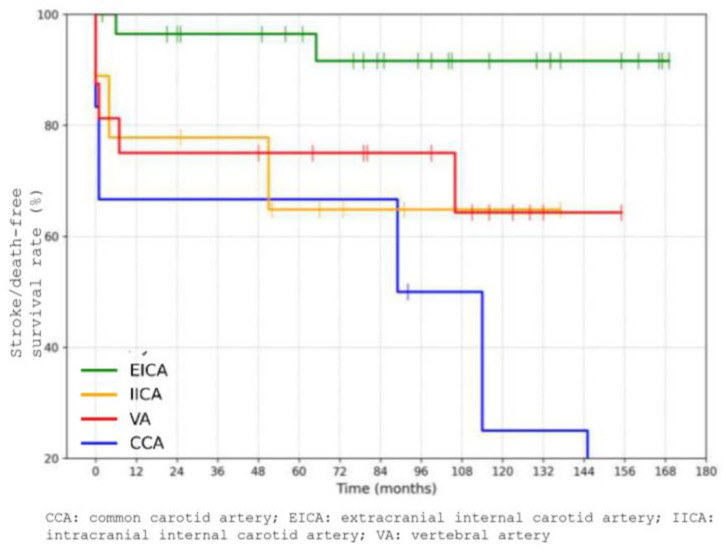
Estimated 10-year stroke/death-free survival rate comparing location of dissections.

A significant difference in outcomes was observed based on the anatomical location of the dissection. Patients with common carotid artery dissections (n = 6, 10%) had worse outcomes, with a 10-year stroke/death-free survival rate as low as 25%, compared to patients with dissections in other cervical arteries (*p* = 0.001) (Figure 7). Among these six patients, one had an associated aortic dissection, three had dissections of the supra-aortic trunks, and two had isolated CCA dissections. One of these dissections was trauma-related. Among the 6 patients with common carotid artery dissections, 4 were treated with antiplatelet therapy, 1 with anticoagulation, and 1 underwent carotid endarterectomy. During follow-up, one patient died from stroke, while the remaining deaths in this subgroup were due to non-neurological causes.

## 4. Discussion

In this retrospective single-center study of 62 patients with CAD, we report long-term outcomes over a median follow-up of 78 months (IQR 54–106), with 10-year estimates of survival and stroke/death-free survival. Our findings support the generally favorable long-term prognosis of CAD, particularly in patients managed conservatively with medical therapy. The overall 10-year stroke/death-free survival rate was 70%, with lower event rates observed in patients treated with anticoagulation compared to those on antiplatelet therapy as previously reported, although the difference did not reach statistical significance.

The predominance of spontaneous over traumatic dissections and the high frequency of ischemic presentations in our cohort are consistent with prior studies [10,11]. Approximately two-thirds of patients presented with ischemic events, confirming that stroke and transient ischemic attack remain the most common clinical manifestations of CAD [12]. This distribution reinforces the importance of early identification and aggressive secondary prevention, particularly in younger populations where CAD is often underdiagnosed. Importantly, the presence of ischemic symptoms at presentation was associated with a lower 10-year stroke/death-free survival compared to non-ischemic presentations, although this difference also did not reach statistical significance. Nonetheless, this trend may reflect a greater initial thromboembolic burden in ischemic presentations, which could influence long-term prognosis even after stabilization.

While the overall long-term outlook was favorable, we identified a subgroup with significantly worse outcomes—patients with dissections involving the common carotid artery. Although most patients in our cohort had dissections of the internal carotid or vertebral arteries, 6 patients (10%) had involvement of the common carotid artery. Among them, the 10-year stroke/death-free survival rate was markedly lower at 25%, compared to patients with dissections at other sites (*p* = 0.001). This is a novel and clinically relevant observation, as limited data exist regarding outcomes of common carotid artery dissection specifically [13]. Within this subgroup, one patient had an associated aortic dissection, three had dissections involving the supra-aortic trunks, and two had isolated common carotid artery dissections. These findings suggest that common carotid artery dissection may reflect a more extensive or aggressive vascular pathology, potentially associated with systemic arteriopathy or connective tissue disorders; however, none of the patients in our cohort had a confirmed diagnosis of connective tissue disease. Our mention of this possibility reflects a speculative hypothesis based on the literature and should be considered an avenue for future investigation rather than a finding of this study. Furthermore, common carotid artery dissections may involve a higher risk of progression or embolic complications, or may reflect more advanced atherosclerotic disease. The poor prognosis in this subgroup may therefore be related not only to anatomical location, but also to associated vascular burden or comorbidity.

These results contribute to the ongoing debate regarding the optimal medical management of CAD. The comparative effectiveness of antiplatelet and anticoagulant therapy remains unresolved. In a recent meta-analysis, Pini et al. showed no significant difference between the two treatments in preventing stroke recurrence, a finding echoed in this study, but with a trend favoring anticoagulation [14]. In our cohort, patients treated with anticoagulation showed a numerically higher 10-year stroke/death-free survival (86%) compared to those treated with antiplatelets (67%); however, this difference was not statistically significant and must be interpreted with caution. Treatment allocation was not randomized but rather based on physician judgment, introducing significant confounding by indication. In particular, patients with imaging findings suggestive of higher embolic risk (such as larger intraluminal thrombus, severe stenosis, or pseudoaneurysm) were more likely to receive anticoagulation, whereas antiplatelet therapy was preferred for patients considered lower risk or with higher bleeding susceptibility. This bias likely masked or exaggerated differences between groups, rendering the observed trend uninterpretable. Therefore, our data do not allow any definitive conclusion on the difference between anticoagulant and antiplatelet therapy in long-term outcomes. Our findings should also be interpreted in the context of the clinical reasoning underlying treatment allocation. In our cohort, anticoagulation was more often prescribed for patients with radiological features associated with greater embolic risk, such as larger thrombus burden, severe stenosis, or pseudoaneurysm, whereas antiplatelet therapy was usually chosen in patients with less severe anatomical involvement or when bleeding risk was a concern. This selection bias may have attenuated or obscured outcome differences between groups, as patients with higher-risk dissections were more frequently treated with anticoagulation. These real-world patterns are consistent with recent literature underscoring the heterogeneity in antithrombotic selection and the ongoing uncertainty regarding the optimal strategy [15].

Only a small minority of patients required surgical or endovascular intervention, consistent with current clinical practice favoring conservative medical management [16]. These interventions were reserved for patients with persistent or worsening symptoms, contraindications to medical therapy, or anatomical considerations that precluded effective pharmacologic treatment. This observation aligns with the general consensus that invasive strategies should be limited to selected cases, and that most CAD cases can be managed effectively with medical therapy alone.

The low rate of stroke recurrence (4%) over the follow-up period underscores the effectiveness of long-term medical therapy in stabilizing the condition. This finding is reassuring and supports the safety and durability of medical treatment in most cases. However, the 24% overall mortality rate—despite a low rate of stroke recurrence—highlights the importance of considering comorbidities and non-neurological causes of death in the long-term management of these patients. It also suggests the need for comprehensive follow-up strategies addressing cardiovascular risk factors, lifestyle modification, and surveillance for systemic vascular disease.

### Limitations

This study has several limitations that should be acknowledged. First, the retrospective and single-center design inherently limits the generalizability of the findings to broader populations. The patient cohort may reflect institutional practices, referral patterns, and regional demographics that differ from those in other centers or healthcare systems. Second, the relatively small sample size—particularly in subgroup analyses—reduces the statistical power to detect significant differences between treatment strategies or dissection locations. This limitation is especially relevant when evaluating less common subgroups, such as patients with common carotid artery dissections or those undergoing surgical or endovascular treatment.

Furthermore, treatment allocation was not randomized, and the initial data entry was not prospectively standardized for research purposes, with decisions made at the discretion of the treating physicians based on individual clinical scenarios. This introduces a potential selection bias, as patients with more severe presentations or greater embolic risk may have been preferentially assigned to anticoagulation or invasive procedures. As a result, observed outcome differences between groups must be interpreted with caution.

Additionally, while mortality data were collected through the institutional database, detailed cause-specific mortality could not always be reliably established, limiting the ability to differentiate between vascular and non-vascular deaths. Finally, although all patients had structured follow-up and no loss to follow-up was recorded, imaging protocols and clinical assessments were not standardized across the entire cohort, which may have led to variations in the detection of recurrence or vessel healing.

Despite these limitations, the study provides valuable insights into the long-term outcomes of CAD in a real-world setting, highlighting key trends and identifying high-risk subgroups worthy of further investigation. Prospective, multicenter studies are needed to confirm these findings and better define optimal management strategies.

## 5. Conclusions

In this real-world, single-center study, cervical artery dissection was associated with a favorable long-term prognosis in most patients. Stroke recurrence was uncommon, and conservative medical management proved effective in the majority of cases. These findings support the continued use of individualized, conservative treatment strategies and underscore the safety of long-term follow-up under medical therapy alone, particularly for patients without high-risk anatomical or clinical features.

Despite the reassuring overall prognosis, our study also identified important risk stratifiers that may help refine long-term management. Notably, patients with common carotid artery (CCA) dissections experienced significantly worse 10-year stroke/death-free survival compared to those with dissections involving other cervical arteries. This observation suggests that dissection location plays a key role in long-term outcomes, and CCA involvement may reflect a distinct pathophysiological profile or greater systemic vascular burden. These patients may benefit from closer surveillance and personalized management strategies, though the optimal approach remains to be defined.

Given the limitations of retrospective single-center data, our results should be interpreted with caution. Nevertheless, they contribute valuable real-world evidence to a rare clinical condition and emphasize the need for prospective, multicenter studies to validate these findings and guide evidence-based decisions regarding the selection of antithrombotic therapy, monitoring intervals, and interventional indications in CAD patients.

## Figures and Tables

**Table 1:** Demographic and cardiovascular risk factors.

	N	%
Male	40	65
Hypertension	35	56
Smoke (active)	7	11
Smoke (previous)	10	16
Dyslipidemia	12	19
Diabetes	8	13
Chronic Obstructive Pulmonary Disease	7	11
Coronary Artery Disease	5	8
Chronic Kidney Disease	4	6
	**Median**	**IQR**
Age (years)	58	56–60

**Table 2:** Location of cervical artery dissections.

	N	%
Common carotid artery	6	10
Extracranial internal carotid artery	29	46
Intracranial internal carotid artery	9	14
Vertebral artery	16	25
Basilar artery *	1	2
Bilateral	5	8

* patient with dissection of both vertebral artery and basilar artery.

**Table 3:** Clinical manifestations of CAD.

Non-Ischemic Manifestations	N	%
Neck pain	2	3
Vertigo	5	8
Headache	15	24
Horner’s Syndrome	2	3
**Ischemic Manifestations**	**N**	**%**
TIA	10	16
Minor Stroke	17	27
Major Stroke	16	25

TIA: Transient Ischemic Attack.

## Data Availability

The data presented in this study are available on request from the corresponding author due to ethical reasons.

## References

[1] Engelter S.T., Traenka C., Von Hessling A., Lyrer P.A. (2015). Diagnosis and treatment of cervical artery dissection. Neurol. Clin..

[2] Wawak M., Tekieli Ł., Badacz R., Pieniążek P., Maciejewski D., Trystuła M., Przewłocki T., Kabłak-Ziembicka A. (2023). Clinical Characteristics and Outcomes of Aortic Arch Emergencies: Takayasu Disease, Fibromuscular Dysplasia, and Aortic Arch Pathologies: A Retrospective Study and Review of the Literature. Biomedicines.

[3] Debette S. (2014). Pathophysiology and risk factors of cervical artery dissection: What have we learnt from large hospital-based cohorts?. Curr. Opin. Neurol..

[4] Metso T.M., Debette S., Grond-Ginsbach C., Engelter S.T., Leys D., Brandt T., Pezzini A., Bersano A., Kloss M., Thijs V. (2012). Age-dependent differences in cervical artery dissection. J. Neurol..

[5] Markus H.S., Hayter E., Levi C., Feldman A., Venables G., Norris J., CADISS trial investigators (2015). Antiplatelet treatment compared with anticoagulation treatment for cervical carotid dissection (CADISS): A randomized trial. Lancet Neurol..

[6] Engelter S.T., Traenka C., Gensicke H., Schaedelin S.A., Luft A.R., Simonetti B.G., Fischer U., Michel P., Sirimarco G., TREAT-CAD investigators (2021). Aspirin versus anticoagulation in cervical artery dissection (TREAT-CAD): An open-label, randomised, non-inferiority trial. Lancet Neurol..

[7] Marnat G., Lapergue B., Sibon I., Gariel F., Bourcier R., Kyheng M., Labreuche J., Dargazanli C., Consoli A., Blanc R. (2020). Safety and Outcome of Carotid Dissection Stenting During the Treatment of Tandem Occlusions: A Pooled Analysis of TITAN and ETIS. Stroke.

[8] Latacz P., Simka M., Brzegowy P., Słowik A., Popiela T. (2019). Endovascular management of carotid and vertebral artery dissections with new generation double-mesh stent and protection systems—Single-center early and midterm results. Postepy Kardiol. Interwencyjnej.

[9] Lyden P.D., Lu M., Levine S.R., Brott T.G., Broderick J., NINDS rtPA Stroke Study Group (2001). A modified National Institutes of Health Stroke Scale for use in stroke clinical trials: Preliminary reliability and validity. Stroke.

[10] Lee V.H., Brown R.D., Mandrekar J.N., Mokri B. (2006). Incidence and outcome of cervical artery dissection: A population-based study. Neurology.

[11] Blum C.A., Yaghi S. (2015). Cervical Artery Dissection: A Review of the Epidemiology, Pathophysiology, Treatment, and Outcome. Arch. Neurosci..

[12] Janquli M., Selvarajah L., Moloney M.A., Kavanagh E., O’Neill D.C., Medani M. (2023). Long-term outcome of cervical artery dissection. J. Vasc. Surg..

[13] Zach V., Zhovtis S., Kirchoff-Torres K.F., Weinberger J.M. (2012). Common carotid artery dissection: A case report and review of the literature. J. Stroke Cerebrovasc. Dis..

[14] Pini R., Faggioli G., Lodato M., Campana F., Vacirca A., Gallitto E., Gargiulo M. (2024). Medical and interventional outcome of dissection of the cervical arteries: Systematic review and meta-analysis. J. Vasc. Surg..

[15] Kaufmann J.E., Mayer-Suess L., Seiffge D., Knoflach M., Engelter S.T., Traenka C. (2025). Management of cervical artery dissection: New evidence and future directions. J. Neurol..

[16] Ahlhelm F., Benz R.M., Ulmer S., Lyrer P., Stippich C., Engelter S. (2013). Endovascular treatment of cervical artery dissection: Ten case reports and review of the literature. Interv. Neurol..

